# Mitochondrial phylogeny and distribution of cytoplasmic male sterility-associated genes in *Beta vulgaris*

**DOI:** 10.1371/journal.pone.0308551

**Published:** 2024-09-27

**Authors:** Keishi Kubota, Mion Oishi, Eigo Taniguchi, Akiho Akazawa, Katsunori Matsui, Kazuyoshi Kitazaki, Atsushi Toyoda, Hidehiro Toh, Hiroaki Matsuhira, Yosuke Kuroda, Tomohiko Kubo

**Affiliations:** 1 Research Faculty of Agriculture, Hokkaido University, Sapporo, Japan; 2 Advanced Genomics Center, National Institute of Genetics, Mishima, Japan; 3 Hokkaido Agricultural Research Center, National Agriculture and Food Research Organization, Memuro, Japan; Universidade de Lisboa Instituto Superior de Agronomia, PORTUGAL

## Abstract

Cytoplasmic male sterility (CMS) is a mitochondrial-encoded trait that confers reproductive defects in males but not in females or any vegetative function. Why CMS is so often found in plants should be investigated from the viewpoint of mitochondrial phylogeny. *Beta vulgaris*, including the wild subspecies *maritima* and cultivated subspecies *vulgaris* (e.g., sugar beet), is known to be mitochondrially polymorphic, from which multiple CMS mitochondria have been found, but their evolutionary relationship has been obscure. We first refined the *B*. *vulgaris* reference mitochondrial genome to conduct a more accurate phylogenetic study. We identified mitochondrial single-nucleotide polymorphic sites from 600 *B*. *vulgaris* accessions. Principal component analysis, hierarchical clustering analysis, and creation of a phylogenetic tree consistently suggested that *B*. *vulgaris* mitochondria can be classified into several groups whose geographical distribution tends to be biased toward either the Atlantic or Mediterranean coasts. We examined the distribution of CMS-associated mitochondrial genes from Owen, E- and G-type CMS mitochondria. About one-third of cultivated beets had Owen-type CMS, which reflects the prevalence of using Owen-type CMS in hybrid breeding. Occurrence frequencies for each of the three CMS genes in wild beet were less than 4%. CMS genes were tightly associated with specific mitochondrial groups that are phylogenetically distinct, suggesting their independent origin. However, homologous sequences of the Owen type CMS gene occurred in several different mitochondrial groups, for which an intricate explanation is necessary. Whereas the origin of cultivated beet had been presumed to be Greece, we found an absence of Owen-type mitochondria in Greek accessions.

## Introduction

In modern evolutionary genetics, the idea that some genetic elements are selfish, which is defined as having ‘characteristics enhancing its own transmission relative to the rest of an individual’s genome but neutral or detrimental to the organism as a whole’ [1], is widely accepted and is sometimes invoked as the ultimate factor for explaining the evolution of puzzling characteristics. Mitochondria are cellular organelles with their own DNA molecules that can evolve selfish genetic elements [2]. A class of mitochondrial selfish genes has been associated with the maternal inheritance of mitochondrial DNA; in hermaphrodites, resources for male gamete production are saved to produce female gametes and/or ensure the organism’s survival [3]. Such incentives have been used to explain the evolution of male sterility-inducing genes encoded by mitochondria [4]; this character is called cytoplasmic male sterility (CMS). Animal CMS has been reported [5], but many more instances of this phenomenon have been found in plants [6]. Therefore, male sterility-inducing mitochondria have frequently evolved during plant mitochondrial divergence.

**Fig 1:**
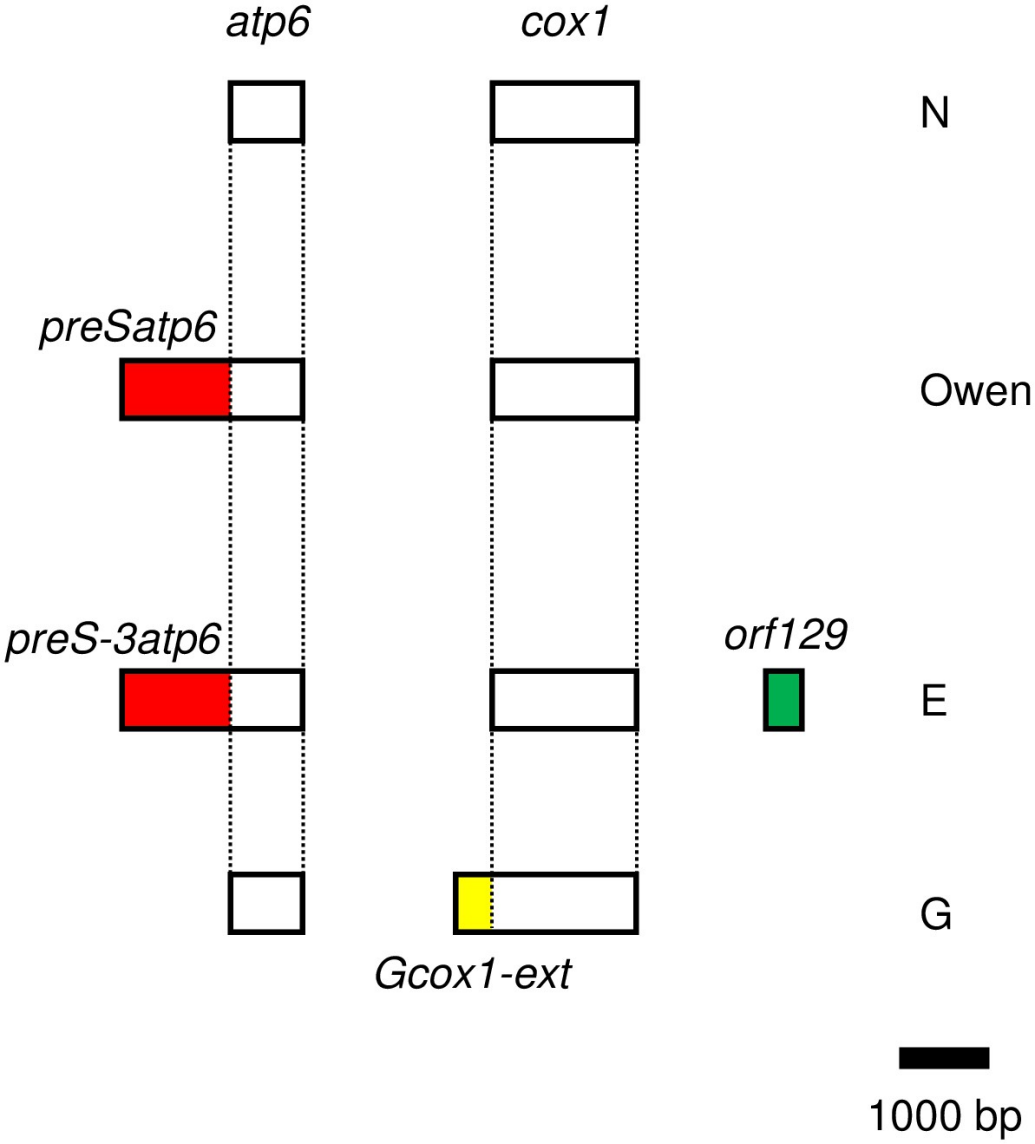
Gene organization of *Beta vulgaris* CMS-associated genes and their orthologues in normal fertile- (N), Owen- (Owen), E- (E), and G-type (G) mitochondria. Boxes identify coding regions. *atp6* and *cox1* are genes encoding a subunit of F0 ATPase and a subunit of cytochrome *c* oxidase, respectively. The direction of transcription is from left to right. Red- and yellow-colored regions identify NH_2_-extensions of *atp6* and *cox1*, respectively. Names of the NH_2_-extensions are shown near the colored boxes. *orf129* is unique to E-type mitochondria, and no homologous sequences are found from other mitochondria. A scale bar is shown in the lower right corner.

*Beta vulgaris*, which includes some crops such as sugar beet, is a species exhibiting high mitochondrial polymorphism [7, 8] with several distinct mitochondria capable of inducing male sterility [9]. Molecular analyses have detailed three male sterility-inducing mitochondrial types (mitotypes): Owen-, E-, and G-types (the latter two are also referred to I-12CMS(3) and FR 4–31, respectively) (references are below). Forrest V. Owen first identified a CMS plant from the sugar beet cultivar ‘US-1’ [10]. Since then, this mitotype (Owen type) has been widely used for hybrid seed production of sugar beets and garden beets [11, 12]. The genes and open reading frames in Owen-type mitochondrial DNA were scrutinized, and an amino-terminal extension (NH_2_-extension) of an *atp6* ORF was identified as producing a specific polypeptide by Owen-type mitochondria [13]. The entire *atp6* gene with the NH_2_-extension is transcribed and translated, after which the precursor polypeptide is separated into two independent polypeptides, mature ATP6 and the NH_2_-extension polypeptide [13]. Thus, the NH_2_-extension seems to be a leader peptide. However, the NH_2_-extension polypeptide is not degraded but is detected in mitochondrial membrane fractions as a homo-oligomer [13]. Because this NH_2_-extension polypeptide is the molecular target of the nuclear suppressor gene for Owen-type CMS [14], this NH_2_-extension is associated with Owen-type CMS (hereafter, this NH_2_-extension is referred to as *preSatp6*; Fig 1).

An ORF termed *orf129* was found in E-type mitochondria [15] (Fig 1). Translation products of *orf129* are detected in membrane and matrix fractions and can potentially induce male sterility because transgenic plants expressing a fusion protein of ORF129 with a mitochondrial transit peptide exhibited male sterility [15]. Interestingly, E-type mitochondria encode an *atp6* whose translation product has a long NH_2_-extension whose nucleotide sequence is similar but different from *preSatp6* (hence it was termed *preS-3atp6*; Fig 1) [15–17]. Unlike *preSatp6*, the translation product of *preS-3atp6* was less abundant in flower buds [15].

Reduced Complex IV (cytochrome *c* oxidase) capacity was detected in beets with G-type mitochondria [18]. Among several genes with missense or nonsense mutations [17], *cox1* was identified as the cause of reduced Complex IV capacity [19]. The G *cox1* has an NH_2_-extension that is entirely different from *preSatp6* or *preS-3atp6* [19]. Unlike *preSatp6*, the NH_2_-extension persisted in the mature COXI protein in G mitochondria [19]. Hereafter, the NH_2_-extension of G *cox1* is referred to as *Gcox1-ext*.

The occurrence of multiple CMS mitochondria in a species raises the question of whether each has evolved independently (i.e., each has its own lineage) or whether the various forms have occurred sequentially by modifying a predecessor (i.e., a lineage has produced multiple CMS mitochondria). Whereas each of the three *B*. *vulgaris* CMS mitochondrial types has its own CMS-associated gene, the evolutionary relationship among these mitochondria is still unclear. That is, two possibilities for the evolution of multiple CMS mitochondria are equally supported by previous studies [8, 17, 20]. The opposite conclusions were made from a limited quantity of samples and data, which could be overcome by recent advances in nucleotide sequencing technology, such as Next Generation Sequencing (NGS). On the other hand, using NGS to analyze plant mitochondrial polymorphism needs some caution. Plant mitochondrial genomes are large, typically in the range of 200–700 kbp [21], and the copy number of mitochondrial DNA (mtDNA) per cell is much smaller than those of animal mtDNA and plastid DNA (ptDNA) [22, 23]. Because plant mtDNA has homologous sequences to nuclear- and ptDNA [e.g., 24], it is possible that sequence reads derived from plastid- and nuclear DNA are erroneously mapped onto the reference mitochondrial genome sequence. Exotic reads with slightly different nucleotides from the reference will cause a complex result. Because the repertoire of nuclear DNA homologous to mtDNA (NUMT) exhibits intraspecific polymorphism at a very high level, i.e., enough to cause apparent lineage-specific heteroplasmy [25], heteroplasmic sites should not be included in mitochondrial phylogenetic studies.

*B*. *vulgaris* consists of three intercompatible subspecies: two wild subspecies, ssp. *maritima* and ssp. *adanensis*, and ssp. *vulgaris*, a subspecies of cultivated beet such as leaf-, fodder- and garden beets, in addition to sugar beet [26]. Several nuclear DNA polymorphism studies agreed that the wild subspecies can be separated into two fairly distinct groups, the Mediterranean and the Atlantic groups, which can be further divided into several subgroups [27–31]. Sugar beet nuclear DNA most resembles Greek wild beet nuclear DNA, suggesting a potential origin of cultivated beet [29]. The three *B*. *vulgaris* CMS mitochondrial types have been found in ssp. *vulgaris* and ssp. *maritima* [32, 33]. The Owen-type CMS mitochondria have been found in all types of cultivated beets, including sugar beet [33], garden beet [34], fodder beet [35], and leaf beet [36]. It is unknown whether the CMS mitochondria identified in cultivated beets can be traced back to the very beginning of domestication or are the result of later introgression from crosses between cultivated and wild beets. Distribution of the CMS mitochondria in ssp. *maritima*, the ancestral taxon of cultivated beets, seems central to addressing this question and assessing the possibility of introgression.

In this study, we classified and inferred the phylogeny of *B*. *vulgaris* mitochondria based on single nucleotide polymorphisms (SNPs) of 600 accessions using NGS data. We also identified accessions with *preSatp6*, *orf129* and *Gcox1-ext* sequences. Based on these data, we inferred the evolution of beet CMS relative to the mitochondrial divergence in this species. We favor the following notions: (1) the distribution of mitochondrial types tends to be geographically biased (i.e., Atlantic and Mediterranean coasts), (2) each of the CMS mitochondria has evolved independently, although there is a puzzling distribution pattern that several phylogenetically remote mitotypes share a unique nucleotide sequence, and (3) the absence of Owen-type mitochondria from Greek wild beet accessions suggests introgression as the origin of Owen-type mitochondria in cultivated beets.

## Materials and methods

### Plant materials

Sugar beet (*B*. *v*. ssp. *vulgaris*) lines NK-195BRmm-O, TA-33BB-CMS(Owen) and TA-33BB-CMS(G) have non-sterility inducing (N)-, Owen-type CMS- and G-type CMS mitochondria, respectively [25, 37], all of which were developed by the Hokkaido Agricultural Research Center, National Agriculture and Food Research Organization, Japan. *B*. *macrorhiza* was included as an outgroup taxon because it is classified into the *Corollinae* section, whereas *B*. *vulgaris* is in the *Beta* section [26]. A *B*. *macrorhiza* accession, Ames 4511, was obtained from the United States Department of Agriculture. Plants were grown in a greenhouse at Hokkaido University.

### Genome sequencing

Genomic DNA libraries were constructed from the green leaves of TA-33BB-CMS(Owen), TA-33BB-CMS(G), and Ames 4511 following the procedures described in [25] using a TruSeq DNA PCR-Free Library Prep Kit (Illumina, San Diego, CA, U.S.A.)(fragment size was 350 bp). A Novaseq 6000 (Illumina) was used for paired-end sequencing (150 bp). The numbers of total reads and total read bases were 153852302 and 23 Gbp (TA-33BB-CMS(Owen)), 223452846 and 33 Gbp (TA-33BB-CMS(G)), and 128138268 and 19 Gbp (Ames 4511). Reads for NK-195BRmm-O were reported in [25]. Raw data quality was checked and trimmed as described in [25].

### Resequencing of mitochondria

Sequence reads were mapped onto the sugar beet mtDNA sequence (DDBJ/ENA/NCBI accession number BA000009) following the procedures of [25]. Nucleotides were visualized by igvtools implemented by IGV (https://software.broadinstitute.org/software/igv/) [38] for manual correction.

### Raw data for variant calling

In addition to our data, short-read sequencing data of *B*. *vulgaris* accessions reported by [29] were retrieved from a public database (S1 Table). Although BETA 6, BETA 7 and BETA 591 are described as belonging to *B*. *macrocarpa* in the passport data of The Leibniz Institute of Plant Genetics and Crop Plant Research, the sequences are likely to be from *B*. *vulgaris* according to [29]. Hence, these sequences are considered to be from *B*. *v*. *ssp*. *maritima* in the present study. The data were downloaded using the prefetch and the fastq-dump functions implemented by SRA-Toolkit (https://github.com/ncbi/sra-tools/wiki/01.-Downloading-SRA-Toolkit). We noticed that the coverage of the downloaded data was different from our data. Therefore, we randomly sampled 1.0 x 10^7^ reads from our data to reconstruct data sets with coverage similar to the downloaded data. This procedure was accomplished using seqkit (https://bioinf.shenwei.me/seqkit/download/) [39].

### Mitochondrial variant calling

The raw data were subjected to a quality check by FASTQC (https://www.bioinformatics.babraham.ac.uk/projects/fastqc/), then trimmed using Trimommatic (https://github.com/usadellab/Trimmomatic) [40] with the parameters of LEADING:20, TRAILING:20, MINLEN:80, and SLIDINGWINDOW:5:20. The resultant sequence reads were mapped onto a reference mitochondrial genome that was resequenced in this study (see above) using the Burrows-Wheeler Aligner (BWA)-mem (http://bio-bwa.sourceforge.net/) [41] with default parameters. A SAM file was converted into a BAM file, then sorted and indexed using samtools (http://www.htslib.org/) [42]. Using igvtools, we expressed the results of read mapping as text files in which the nucleotide composition of each site was recorded. The text files were manipulated with Microsoft Excel (Microsoft Japan, Tokyo, Japan) to calculate each site’s read depth and the frequency of consensus nucleotides. We noticed three classes of read depth at each site of an accession, namely ~3000, ~100 and ~5. Because the copy number of ptDNA per cell is much higher than mtDNA, the ~3000 depth class is the result of mapping ptDNA reads onto the mitochondrial reference due to homologous sequences in mtDNA and ptDNA. As sequences specific to a mitochondrial type are known in *B*. *vulgaris* (e.g., a total of 28.5 kbp sequences in N-type CMS mitochondria are absent from Owen-type mitochondria [43]), NUMTs with such specific sequence origin look like low-copy-number mtDNA molecules in our plant mitochondrial NGS study. Thus, the ~5 depth class is the result of mapping NUMTs onto the mitochondrial reference, but the accessions’ mitochondria do not have the corresponding sequences. To exclude the nucleotides of non-mitochondrial DNA origin, sites with read depths equal to or less than 20 and equal to or more than 900 were filtered out, and these sites were considered missing data. Of the remaining sequences, sites occupied by multiple nucleotides may represent true heteroplasmy, but they can occur because of mapping NUMT, mapping repeated sequences, or other erroneous mapping. In this study, such sites were considered missing data for which consensus nucleotides with frequencies equal to or less than 80% were filtered out. The remaining nucleotide sequences were aligned in Excel to find SNPs. In the alignment file, sites with missing data, an indel, or no SNP were filtered out. The residual nucleotides are reported in a multi-FASTA-format file.

### Principal component analysis

FASTA-formatted SNPs sequences were converted into the VCF format using SNP-sites (https://github.com/sanger-pathogens/snp-sites) [44]. Tassel 5 software (https://tassel.bitbucket.io/) was used for principal component analysis (PCA) [45].

### Hierarchical clustering analysis

Hierarchical clustering analysis used the linkage function in the hierarchy module of the cluster subpackage in SciPy software (https://docs.scipy.org/doc/scipy/reference/generated/scipy.cluster.hierarchy.linkage.html#scipy.cluster.hierarchy.linkage) that is based on Bray-Curtis distance metrics using an average linkage method. A dendrogram was visualized by the dendrogram function of the same module (https://docs.scipy.org/doc/scipy/reference/generated/scipy.cluster.hierarchy.dendrogram.html#scipy.cluster.hierarchy.dendrogram).

### Phylogenetic tree construction

MEGAX software (https://www.megasoftware.net/) was used to construct a tree using the maximum likelihood method (bootstrap iteration was 1000) [46]. The tree was visualized using ITOL (https://itol.embl.de/) [47].

### Searching for specific sequences

Mitochondrial contigs were constructed using Getorganelle software (https://github.com/Kinggerm/GetOrganelle) [48]. Queries for BLAST searches (https://blast.ncbi.nlm.nih.gov/Blast.cgi) were retrieved from DDBJ/ENA/NCBI databases using accession numbers BA000024 (*preSatp6*), AB355937 (*orf129*), and FP885871 (*Gcox1-ext*).

## Results

### Refining the *B*. *vulgaris* mitochondrial reference

As we pointed out earlier [25], the reference mtDNA sequence for sugar beet (DDBJ/ENA/NCBI accession number BA000009) was constructed using an old breeding line. This sequence may contain sequencing errors since it was constructed using an old sequencing technology [49]. To solve this issue, we conducted an NGS analysis of a modern variety (NK-195BRmm-O) and mapped the short reads onto the reference to assemble a single continuous sequence. Taniguchi et al. [25] conducted a similar experiment and found many intra-individual polymorphic sites that contained two or more nucleotides in a site, although the DNA sample was isolated from a single plant. We encountered a similar phenomenon in the present study and devised a strategy to avoid ambiguity in the nucleotides in such sites. As we identified plastid DNA-homologous sequences on the reference sequence [25], we considered that alleles with an extremely high depth (generally more than 10000) mapped onto a plastid DNA-homologous region were artifacts of cpDNA origin and were not included in the new reference. Of the other alleles, those with lower depth were excluded because they are likely derived from NUMT or represent heteroplasmy. As a result, NK-195BRmm-O mtDNA was shown to be 368963 bp in length, which differed from the registered reference at 260 sites. We used the NK-195BRmm-O mtDNA sequence as the reference for the following studies.

### Single nucleotide polymorphism of *B*. *vulgaris* mitochondria

We analyzed the mitochondrial polymorphism in *B*. *vulgaris* using the NK-195BRmm-O mtDNA sequence. The sequence data used in this study included 297 *B*. *v*. ssp. *vulgaris-*, 273 *B*. *v*. ssp. *maritima*, and 29 *B*. *v*. ssp. *adanensis* accessions. An accession of *B*. *macrorhiza* was added as an outgroup taxon. Thus, a total of 600 accessions were used. We called mitochondrial variant nucleotides from the shared sequences among all samples. As a result, we identified 749 SNP sites, with 2.03 alleles per site on average.

**Fig 2:**
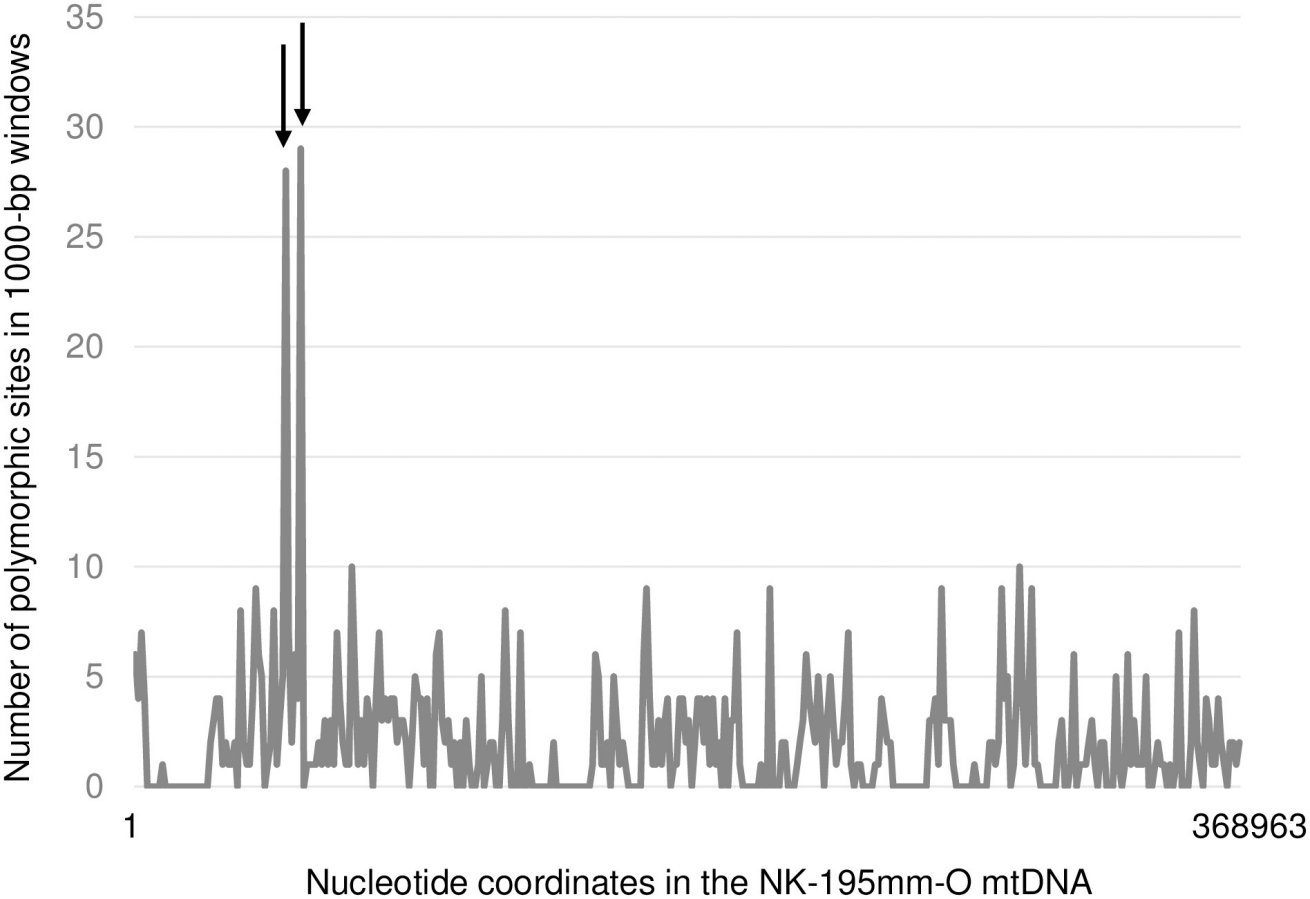
Distribution of SNP sites in the sugar beet mitochondrial genome. Horizontal and vertical axes indicate the NK-195BRmm-O reference genome and the number of polymorphic sites in 1000-bp windows, respectively. Two arrows depict the two windows with the most polymorphic sites.

The number of polymorphic sites in every 1000 bp window in the reference mitochondrial genome is shown in Fig 2. In general, the sites were distributed evenly, and the numbers ranged from zero to ten, but two windows (nucleotide position 50001–51000 and 55001–56000 in the NK-195mm-O reference) were exceptional because these polymorphic sites totaled 27 and 29 sites, respectively.

### Phylogeny of *B*. *vulgaris* mitochondria

**Fig 3:**
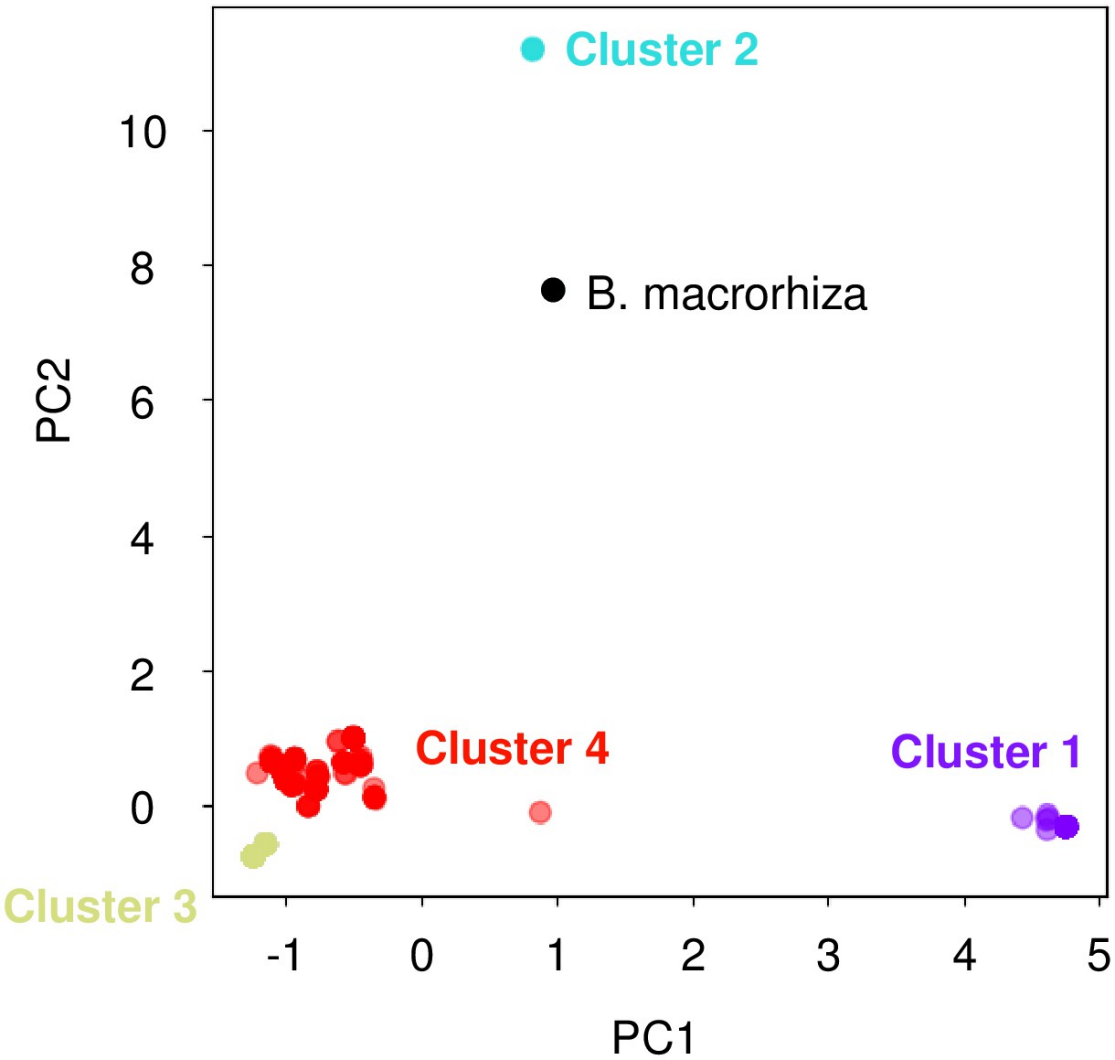
PCA scatter plot of *B*. *vulgaris* mitochondria calculated based on SNPs. Horizontal and vertical axes indicate PCA1 and PCA2, respectively. The dot colors correspond to clusters in the dendrogram shown in Fig 4; purple, Cluster 1; turquoise, Cluster 2; yellow-green, Cluster 3; and red, Cluster 4. A black dot indicates *B*. *macrorhiza*.

We took two approaches to analyze the polymorphism of *B*. *vulgaris* mitochondria. PCA used SNPs from 749 sites. The cumulative proportion explained exceeded 0.9 at PC7 (S2 Table). These results suggest the separation of *B*. *vulgaris* mitochondria into three or more groups (Fig 3).

**Fig 4:**
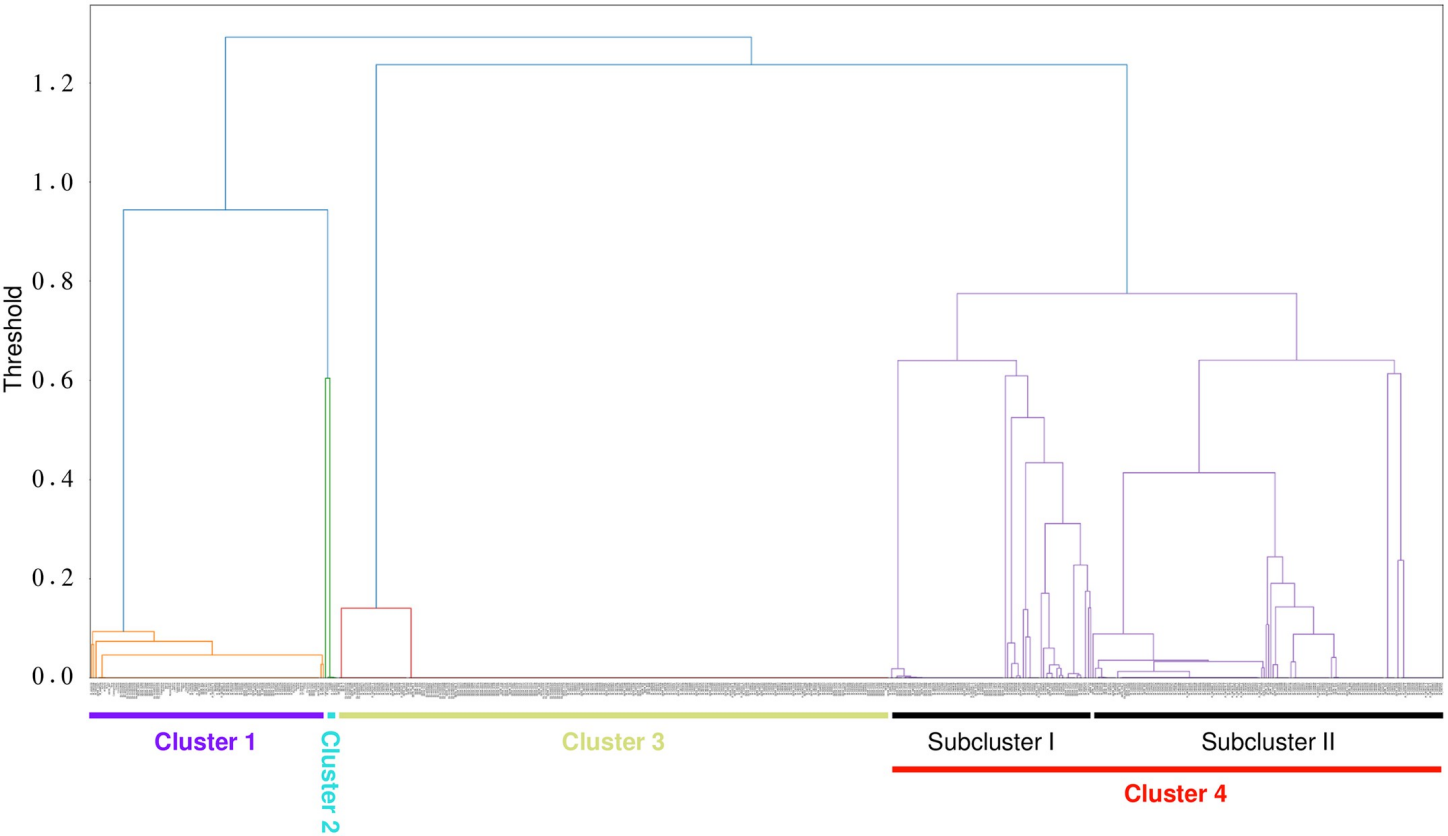
Dendrogram of *B*. *vulgaris* mitochondrial genomes based on hierarchical clustering analysis. The original image with accession names is provided as S1 Fig. Clusters 1 to 4 and Subclusters I and II are indicated by horizontal lines.

We next calculated similarities between all pairs of the accessions and conducted a hierarchical clustering analysis to draw a dendrogram (Fig 4). Together with the results of the PCA, we separated the mitochondria into four clusters: Cluster 1 had a low level of intra-cluster polymorphism and contained TA-33BB-CMS(Owen). Cluster 2 was the smallest cluster, with only five accessions. TA-33BB-CMS(G) was a member of Cluster 2. Cluster 3 is another group with very low polymorphism to which NK-195BRmm-O belonged. Cluster 4 was, in contrast, rather polymorphic. Hence, this cluster appears as a miscellaneous group. Cluster 4 may be further separated into Subclusters I and II.

**Fig 5:**
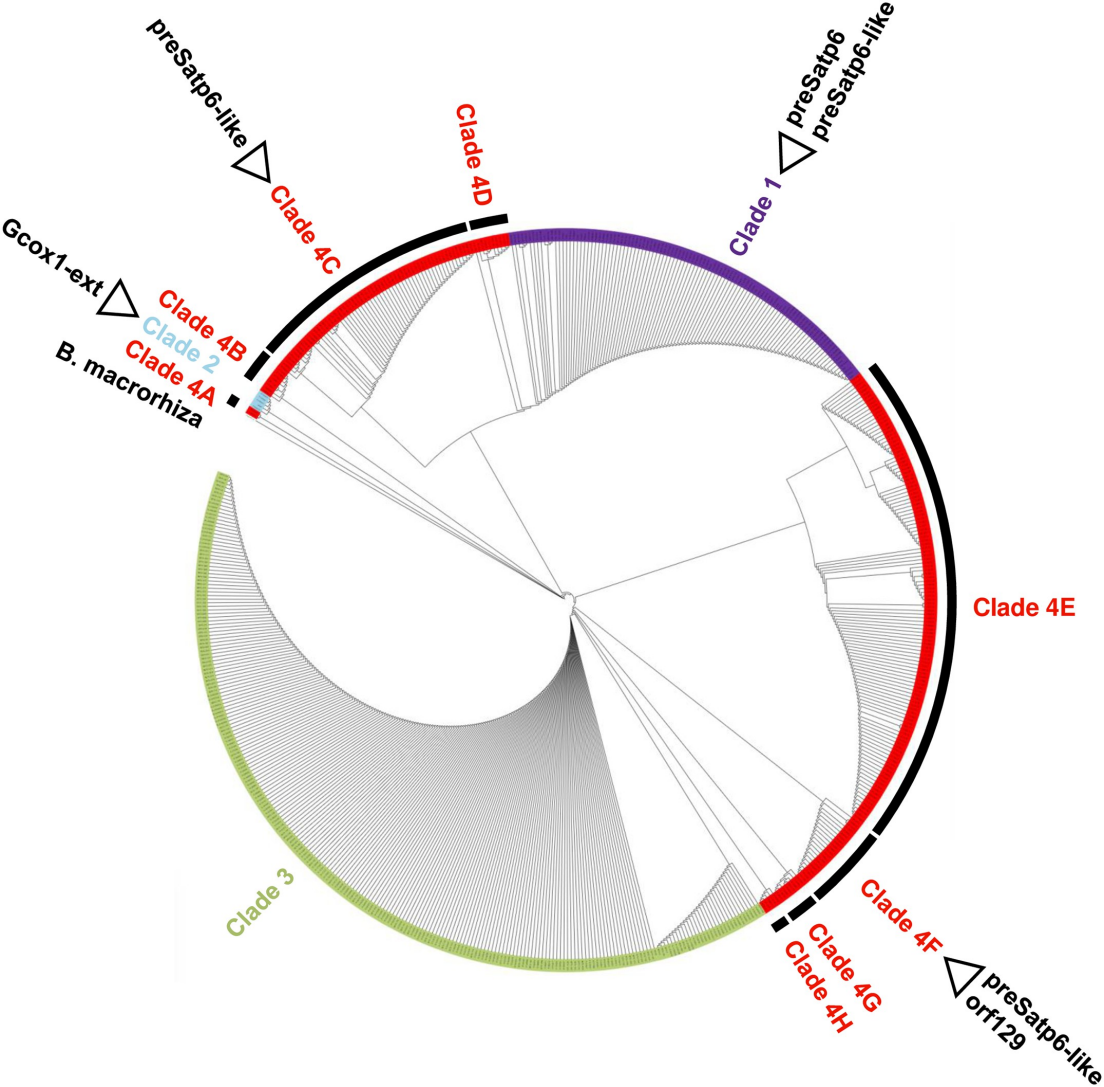
A phylogenetic tree of *B*. *vulgaris* mitochondria. The tree was constructed using the maximum likelihood method. The original image with accession names is provided as S2 Fig. Accessions are highlighted in the same colors as shown for their Clusters in Fig 4: purple, Cluster 1; turquoise, Cluster 2; yellow-green, Cluster 3; and red, Cluster 4. Names of the Clades are shown outside the circle. Arcs show the extent of Clades 4A to 4H. *B*. *macrorhiza* is shown as an outgroup. Open triangles indicate clades with accessions having *preSatp6*, *preSatp6*-like, *orf129*, or *Gcox1-ext*.

We used our polymorphic data to construct a phylogenetic tree (Fig 5). Several clades appeared in the tree, and we investigated whether they corresponded to the clusters on the dendrogram identified by hierarchical clustering analysis. Accessions of Clades 1, 2, and 3 corresponded to Clusters 1, 2, and 3, respectively. Hence, these clades were equivalent to the clusters. There seemed to be two subclades in Clade 3, a result also suggested in the dendrogram (see Fig 4). In contrast, the other accessions were scattered in different branches of the tree, and the Cluster 4 accessions did not form a single clade. We named these clades as Clades 4A to 4H and found that accessions of Clades 4A to 4D corresponded to Subcluster I, whereas those of Clades 4E to 4H corresponded to Subcluster II.

Mitotypes in each subspecies were investigated. Of the 297 accessions of ssp. *vulgaris*, 92 and 185 accessions were Clades 1 and 3 types, respectively. The remaining 20 accessions were Clades 2 (one accession), 4B (two accessions), 4C (13 accessions), 4D (one accession), 4F (two accessions), and 4G (one accession). Subspecies *maritima* was found in all the clades, whereas ssp. *adanensis* did not form a unique clade but coexisted with *B*. *v*. ssp. *maritima*.

To investigate the geographical distribution of mitotypes, we focused on the two wild subspecies (*B*. *v*. ssp. *maritima* and ssp. *adanensis*). We consulted passport data to check the collection sites for these accessions to determine if there was any relationship with a cluster or clade. Collection sites were simply classified into Atlantic and Mediterranean coasts. When the clades were separated into two large groups as Clades 1+2+4A+4B+4C+4D and Clades 3+4E+4F+4G+4H (see Fig 5), the Mediterranean and Atlantic accessions, respectively, were overrepresented (Table 1A; *p* = 2.97 x 10^−19^; Fisher’s exact test). It should be noted that this trend was not always clear when a single clade was investigated; for example, there were five Clade 1 accessions from the Atlantic coast, and seven from the Mediterranean coast, and 25 Clade 3 accessions from the Atlantic coast, and 32 from the Mediterranean coast (Table 1B).

**Table 1:** Number of wild beet accessions with different mitochondrial types.

A. Number of accessions in two large mitochondrial groups												
	Group											Total
	Clades 1+2+4A+4B+4C+4D						Clades 3+4E+4F+4G+4H					
Atlantic coast	12						153					165
Mediterranean coast	70						61					131
Total	82						214					296
B. Distribution of accessions into clades												
	Clade											Total
	1	2	3	4A	4B	4C	4D	4E	4F	4G	4H	
Atlantic coast	5	3	25	2	2	0	0	107	17	4	0	165
Mediterranean coast	7	1	32	0	4	49	9	18	3	3	5	131
Total	12	4	57	2	6	49	9	125	20	7	5	296

### Distribution of CMS-associated sequences

We searched for accessions having beet CMS-associated genes, *preSatp6*, *orf129* and *Gcox1-ext* (Fig 1) and used the sequences as queries for BLAST searches. We obtained mitochondrial contigs from 481 accessions for this analysis.

When *preSatp6* was used as a query, one hundred accessions were positive. We classified these accessions into two groups based on their homology to the query. One group showed high homology to *preSatp6* as the alignment length was 1161 bp, the same length as *preSatp6*, and the number of mismatches was three or less. We consider these 87 accessions (Table 2) to have *preSatp6*, all belonging to Clade 1 (Fig 5 and S1 Table). The other *preSatp6* positive accessions were considered not to have *preSatp6* but to have homologues of *preSatp6* because the alignment length was less than 1161 bp with eight or more mismatches. We referred to such *preSatp6* homologues as *preSatp6*-like. Thirteen accessions had *preSatp6*-like sequences and belonged to Clade 1 (one accession), Clade 4C (one accession) and Clade 4F (11 accessions) (Fig 5 and S1 Table). Of the 262 cultivated beet (*B*. *v*. ssp. *vulgaris*) accessions whose mitochondrial contigs were available, 80 accessions had *preSatp6* sequences, whereas seven of the 218 wild beet (*B*. *v*. ssp. *maritima* and *B*. *v*. ssp. *adanensis*) accessions had *preSatp6*, all of which were *B*. *v*. ssp. *maritima* (Table 2 and S1 Table). *B*. *v*. ssp. *maritima* had 13 accessions with *preSatp6*-like sequences (Table 2).

**Table 2:** Number of accessions having CMS-associated genes of *B*. *vulgaris* mitochondria.

	Cultivated beets (*B*. *vulgaris* ssp. *vulgaris*)	Wild beets (*B*. *vulgaris* ssp. *maritima*)		Total
		Atlantic coast	Mediterranean coast	
*preSatp6*	80	7		87
		4	3	
*preSatp6*-like	0	13*^1^		13
		9	3	
*orf129*	0	9		9
		8	1	
*Gcox1-ext*	1	1		2
		1	0	
Total	81	30*^1^		111
		16	13	

*^1^ The collection site of one accession is unknown

Nine accessions contained *orf129* (Table 2). All were *B*. *v*. ssp. *maritima* that belonged to Clade 4F (Fig 5 and S1 Table). All nine accessions were also positive to *preSatp6*-like (S1 Table). Four accessions were *preSatp6*-like positive but *orf129* negative (S1 Table).

*Gcox1-ext* was found in one *B*. *v*. ssp. *maritima* and one *B*. *v*. ssp. *vulgaris* accession (Table 2). The latter is TA-33BB-CMS(G), a sugar beet line to which we introduced G-type mitochondria [37]. These two accessions belonged to Clade 2 (Fig 5 and S1 Table). Mitochondrial contigs were obtained from only two of the five Clade 2 accessions because of the low coverage of reads in the original sequence data.

We checked the geographic distribution of the accessions to determine whether the collection site was the Atlantic or Mediterranean coast. As shown in Table 2, the *preSatp6-*, *preSatp6*-like- and *orf129*- positives were found from both geographic areas. The *orf129-* and *preSatp6*-like positives appeared more frequently in the Atlantic coast samples, but this result is inconclusive. In the previous section, clades were classified into Atlantic overrepresented and Mediterranean overrepresented groups. The *orf129*-positive accessions belong to the Atlantic coast group, whereas the *preSatp6* positive and *Gcox1-ext* positive accessions belong to the Mediterranean coast group.

## Discussion

*B*. *vulgaris* mitochondria are highly polymorphic [8, 32, 36], but their relationships were previously ambiguous. Nuclear genome analyses have provided insights into the evolution and domestication of this species [27–29, 50]. Our results complement these nuclear genome studies because the mode of mitochondrial inheritance is different (i.e., biparental vs. maternal). Our study was based on mitochondrial SNPs identified as occurring at 2.03 SNPs per 1000 bp. Our procedure to identify SNPs was very conservative as no missing data or indel was allowed. Sites with multiple nucleotides (i.e., intraindividual polymorphic sites) are very frequent in the NGS analysis of plant mitochondria, but we did not consider such sites as representing heteroplasmy [cf., 25]. Therefore, we counted only major nucleotides. Most identified SNP sites were evenly distributed throughout the genome; however, exceptionally high densities were detected in two regions (50001–51001 and 55001–56000 on the reference genome) containing *ccmC* and *atp4-nad4L*, respectively. Sugar beet *ccmC* (cytochome C maturation) is unique as its ORF is extended with a 5’ terminus encoding an extra NH_2_ polypeptide [51]. The extended ORF is translated and may act as a leader peptide as it is not included in the mature CCMC protein [51]. Therefore, any indel that induces a frameshift mutation is prohibited because it precludes translation of the downstream CCMC protein. However, nucleotide substitutions may be allowed if they do not impair CCMC protein function. This observation explains the enrichment of SNPs without indels in this region. There were 27 SNPs in this region, of which 26 were found in the outgroup accession *B*. *macrorhiza*, a result consistent with its phylogenetic relationship. Linkage of *atp4-nad4L* (encoding a subunit of F0 ATPase and a subunit of NADH dehydrogenase, respectively) is conserved in some dicotyledonous plants [52], suggesting an evolutionary force to maintain this association. In plant mitochondrial genomes, the mutation rate is consistent throughout the genome, but selection guides the level of polymorphism [53]. Considering this observation, why indels are prohibited in the *atp4-nad4L* region but not substitutions is an interesting but unresolved question.

Based on our SNP analyses, we propose that the *B*. *vulgaris* mitochondrial genomes can be grouped into four Clusters, 1 to 4. Cluster 4 is more polymorphic than the others and can be subdivided into two subclusters. This finding is reflected in the phylogenetic tree in which Cluster 4 accessions are scattered to form distinct clades, with the clades forming two subclusters.

*B*. *v*. ssp. *maritima* is the most polymorphic taxon in terms of its mitochondrial sequences because accessions of this subspecies are represented in all four Clusters. The other subspecies are less polymorphic compared to ssp. *maritima*. Most of the accessions of ssp. *adanensis* belong to Cluster 4, but BETA 1262 and BETA 1693 are exceptions that were classified into Clusters 1 and 3, respectively. Although the taxon of these accessions is described as ssp. *adanensis* in their passport data, Wascher et al. [29] proposed that these accessions are ssp. *maritima* based on the phylogeny of their nuclear DNA. Our data support this notion from the viewpoint of mitochondrial DNA. The other ssp. *adanensis* accessions belong to either Clade 4C or 4D, clades exclusive in the Mediterranean coast (Table 1B). As both the Clades also include ssp. *maritima*, the mitochondrial divergence between ssp. *adanensis* and ssp. *maritima* is under detectable in our present study. This contrasts with the results of the nuclear DNA study by Wascher et al. [29], which showed that ssp. *adanensis* formed its own clade in the phylogenetic tree. We have no clear explanation to these contrasting results but the split of the two subspecies might have been so recent that the time to accumulate enough mutations would have been insufficient for plant mitochondria whose rate of point mutation is very low compared to nuclear genome [21]. It should be noted that this study focuses only on mitochondrial point mutations.

A vast majority of the ssp. *vulgaris* accessions used in this study were predominantly sugar beets that originated from selections of fodder beets in the late 18th century [54]. Hence, a severe bottleneck effect is expected that reduced the variation of mitochondrial genome organization in this crop [35]. Additionally, current sugar beet cultivars are developed by hybrid breeding using Owen-type mitochondria [11]. This information explains why accessions of ssp. *vulgaris* very frequently appeared in Clusters 1 and 3. The 20 ssp. *vulgaris* accessions, which were sugar-, leaf- and an unknown type of beet, seem to be outliers as their mitochondria were classified to Cluster 4. The mislabeling of genetic resources can explain this observation, but another possibility is the introgression of wild beet mitochondria. Goldman and Navazio [12] pointed out that the introgression of other beet types can explain the morphological polymorphism of garden beet during dissemination of this crop in Europe. Kanomata et al. [55] detected wild beet-like mitochondria from a minor fraction of garden-beet genetic resources. Such introgression may have occurred in other beets, too.

*B*.*v*. ssp. *maritima* comprises Atlantic and Mediterranean coast types [27–31, 56]. This finding has been associated with postglacial dissemination of this subspecies from at least two different glacial refugia in Northwest African- and East Mediterranean coasts, respectively [57]. This trend can be seen in wild beet mitochondria when the clades were separated into two large groups (Table 1). However, after breaking down the large group into clades, the geographical distribution of some clades (e.g., Clade 1 or Clade 3) less followed this notion. A founder effect is insufficient to explain the distribution pattern of *B*. *vulgaris* mitochondria, and some other factors, such as selection, should be considered. Provided that the prevalence of some clades in both the Atlantic and Mediterranean coast reflects the maintenance of ancestral polymophism, a balancing selection could play role in this gynodioecious species. It is interesting to note that a population genetic theory postulated such a balancing selection for maintaining mitochondrial polymorphism in a gynodioecious species that exhibits coexistence of hermaphrodites and females due to the action of CMS in a population [3].

We identified all three CMS-associated mitochondrial genes from *B*. *vulgaris*. *preSatp6* was found in 80 of 262 ssp. *vulgaris*, a result that reflects hybrid breeding of sugar beet using Owen-type mitochondria. Besides ssp. *vulgaris*, the number of accessions having *preSatp6*, *orf129* and *Gcox1-ext* were 7, 9 and 1, respectively. Therefore, the frequency of these CMS-associated genes in wild subspecies is generally low. Note that this is a macroscopic view, and areas with enriched CMS mitochondria are also known [e.g., 58], suggesting a patchy distribution of CMS mitochondria. What creates such distribution patterns is a future question. CMS genes were identified from the Atlantic coast and Mediterranean coast accessions, but examples of *orf129* are more frequent in the former (eight and one, respectively). We identified *Gcox1-ext* from only one Atlantic accession, but a report identifies this mitotype from a Mediterranean coast *B*. *v*. ssp. *maritima* [19]. Our data are insufficient to reach conclusions about the geographical distribution of CMS mitochondria (see the above discussion on mitochondrial balancing selection and CMS).

*preSatp6* was found only in Cluster 1 (the equivalent to Clade 1); in Cluster 1, of 99 accessions in which contigs are available, 95 accessions were categorized as having *preSatp6*. It is an interesting but unresolved question whether the mitochondria of the four accessions lacking *preSatp6* represent intermediates on their way to evolving to Owen-type or a degenerated type. *orf129* was exclusive to Clade 4F. In Clade 4F, a few accessions lacking *orf129* can be found; they may be either intermediate or degenerate forms. *Gcox1-ext* is exclusive to Clade 2, the most remote group from the other clades. As the accessions of Clade 2 are few, it is necessary to investigate additional Clade 2 accessions to identify accessions without *Gcox1-ext*. Given the phylogenetic relationships among Clades 1, 2 and 4F, the three CMS-associated genes obviously evolved independently. This conclusion is very similar to previous studies that are based on the polymorphism of chloroplast DNA segments and mitochondrial DNA segments [8, 20].

Homologous sequences to *preSatp6* were detected in Clades 1, 4C, and 4F. We reported that E-type mitochondria have a homologous but different sequence to *preSatp6*, which we named *preS-3atp6* [15]. Some *preSatp6-*like sequences accompanying *orf129*, the CMS gene associated with E-type CMS mitochondria, may be identical to *preS-3atp6*. In the dendrogram and the phylogenetic tree, no direct relationship was identified between Clade 1 (Cluster 1) and Clade 4F (a part of Subcluster I). Given the maternal inheritance of beet mitochondria, it will be necessary to consider multiple losses or multiple independent lines of evolution of *preSatp6* homologous sequences during the evolution of *B*. *vulgaris* mitochondria to explain this enigmatic distribution pattern. Although recombination between different mitochondrial genomes is a scenario to avoid such a complex explanation, other unknown mechanisms may be involved in beet CMS evolution.

Wascher et al. [29] reported that the nuclear DNA of cultivated beet most resembles that of Greek wild beet, leading them to propose that the origin of cultivated beet was Greece. In our study, cultivated beet mitochondria were composed mainly of accessions in Clades 1 and 3, whereas Greek wild beet accessions were in Clades 3 and 4. That Clade 3 is common to both cultivated beets and Greek wild beets is consistent with the hypothesis of Wascher et al. [29]. On the other hand, Greek wild beets had no Clade 1 mitochondria in our study. As Owen-type mitochondria are the major constituent of Clade 1, our results document the absence of Owen- type mitochondria in Greek wild beet accessions. Hence, the population of originally cultivated beets may not possess Owen-type mitochondria. As Clade 1 mitochondria were identified in accessions from East Mediterranean regions, such as Turkey, further analysis is necessary to draw conclusions. Though at a generally low frequency, Owen-type mitochondria have been identified from leaf-, garden-, and fodder beets [34–36]. Possibly, Owen-type mitochondria were introgressed into some cultivated beets from an unknown source and were maintained until F. V. Owen first identified them from the sugar beet cultivar ‘US-1’ in the 20th century [33].

## Supporting information

S1 Table Accessions used in this study. (XLSX)

S2 Table Eigenvalues and the proportion of variation in the principal component analysis of *B*. *vulgaris* mitochondrial SNPs. (XLSX)

S1 Fig Dendrogram of *B*. *vulgaris* mitochondria based on the hierarchical clustering analysis. Accession names can be seen by magnifying the image to 400–800%.
(PDF)

S2 Fig Phylogenetic tree of *B*. *vulgaris* mitochondria. Accession names can be seen by magnifying the image to 400–800%.
(PDF)
