# Supplementary figures and images for: Mitochondrial phylogeny and distribution of cytoplasmic male sterility-associated genes in Beta vulgaris
Source: PLoS One. 2024 Sep 27;19(9):e0308551. doi: 10.1371/journal.pone.0308551 (PMC11432856; doi:10.1371/journal.pone.0308551)

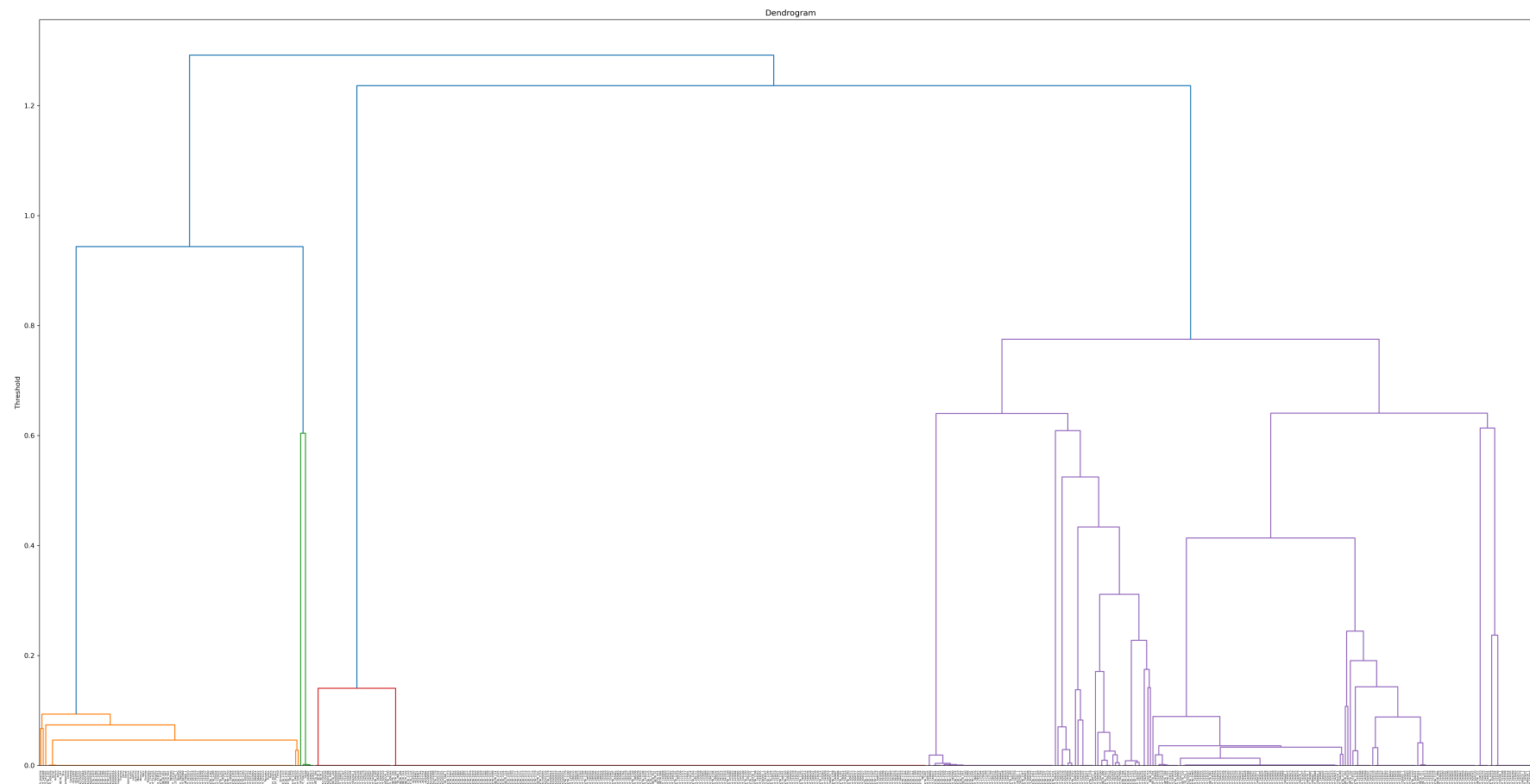

Supplement: S1 Fig — Accession names can be seen by magnifying the image to 400–800%. (PDF) [file pone.0308551.s003.pdf]

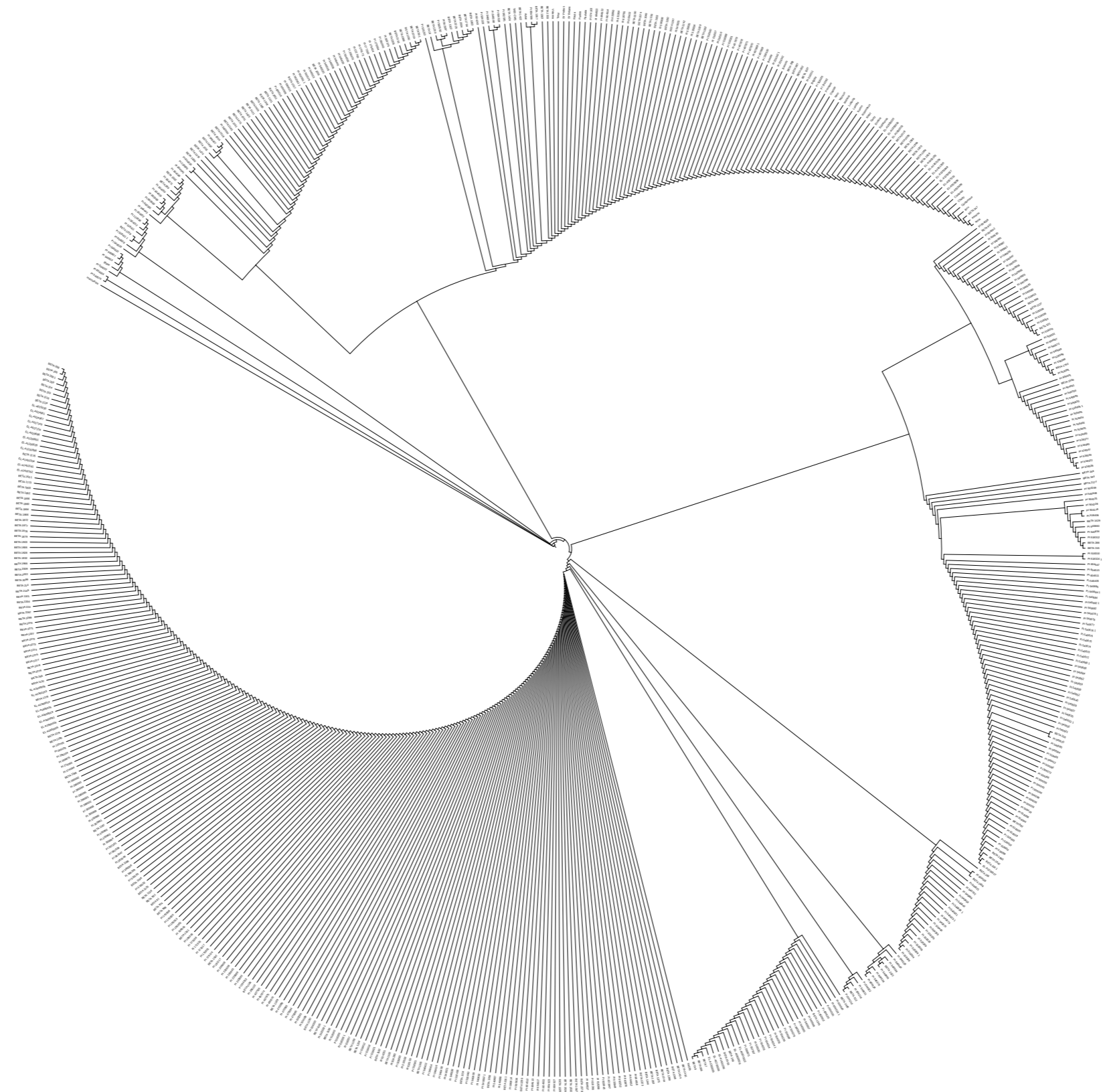

Supplement: S2 Fig — Accession names can be seen by magnifying the image to 400–800%. (PDF) [file pone.0308551.s004.pdf]
